# Deviations of the SLM Produced Lattice Structures and Their Influence on Mechanical Properties

**DOI:** 10.3390/ma15093144

**Published:** 2022-04-26

**Authors:** Radek Vrána, Tomáš Koutecký, Ondřej Červinek, Tomáš Zikmund, Libor Pantělejev, Jozef Kaiser, Daniel Koutný

**Affiliations:** 1 Institute of Machine and Industrial Design, Faculty of Mechanical Engineering, Brno University of Technology, Technická 2896/2, 616 69 Brno, Czech Republic; radek.vrana@vut.cz (R.V.); ondrej.cervinek@vut.cz (O.Č.); daniel.koutny@vut.cz (D.K.); 2 CEITEC-Central European Institute of Technology, Brno University of Technology, Purkyňova 123, 612 00 Brno, Czech Republic; tomas.zikmund@ceitec.vutbr.cz (T.Z.); jozef.kaiser@ceitec.vutbr.cz (J.K.); 3 Institute of Materials Science and Engineering, Faculty of Mechanical Engineering, Brno University of Technology, Technická 2896/2, 616 69 Brno, Czech Republic; libor.pantelejev@vut.cz

**Keywords:** selective laser melting (SLM), lattice structure, shape and dimension analysis, computed tomography (CT), digitization, finite element analysis (FEA)

## Abstract

Selective laser melting (SLM) is an additive manufacturing technology suitable for producing cellular lattice structures using fine metal powder and a laser beam. However, the shape and dimensional deviations occur on the thin struts during manufacturing, influencing the mechanical properties of the structure. There are attempts in the literature to describe the actual shape of the struts’ geometry, however, on a smaller data sample only, and there is a lack of a universal FEA material model applicable to a wider range of lattice structure diameters. To describe the actual dimensions of the struts, a set of lattice structures, with diameters ranging from 0.6 to 3.0 mm, were manufactured using SLM. These samples were digitized using micro-computed tomography (μCT) and fully analyzed for shape and dimensions. The results show large deviations in diameters of inscribed and circumscribed cylinders, indicating an elliptical shape of the struts. With increasing lattice structure diameter, the deviations decreased. In terms of the effect of the shape and dimensions on the mechanical properties, the Gaussian cylinder was found to describe struts in the diameter range of 1.5 to 3.0 mm sufficiently well. For smaller diameters, it is appropriate to represent the actual cross-section by an ellipse. The use of substitute ellipses, in combination with the compression test results, has resulted in FEA material model that can be used for the 0.6 to 3.0 mm struts’ diameter range. The model has fixed Young’s and tangential modules for these diameters and is controlled only by the yield strength parameter (YST).

## 1. Introduction

Selective laser melting (SLM), one of the powder bed fusion (PBF) technologies, is an additive manufacturing technology that allows for the production of metallic components—layer-by-layer—directly from CAD data. The manufacturing process starts by depositing a thin layer of fine metal powder on a base plate. Then, the powder is melted in a protective atmosphere by a laser beam, the base plate is lowered by the one-layer thickness, and the next layer is deposited directly on the previous one. The process is repeated until the whole part is produced [1]. The main advantage of additive production is the possibility to build a complex geometry, such as lattice structures, which cannot be achieved conventionally [2]. Due to their excellent load-to-weight ratio, they have the potential for application in the aerospace or space industries [3].

The lattice structures are usually cellular structures, composed of regularly repeated unit cells. They could have different shapes, but most of them are made of inclined thin struts. Due to the geometry that cannot be manufactured using support structures, a lot of overhanging areas occur. This causes the surrounding powder to be melted on the bottom part of overhanging areas during SLM manufacturing [4], and high surface roughness will appear, along with deviations in the shape and dimensions of the manufactured geometry. These imperfections could be partially eliminated by minimizing used layer thickness [5] and modification of the laser process parameters during SLM manufacturing of lattice structures [6]; however, due to complex lattice geometry, some geometrical deviations between the design (CAD) data and manufactured lattice structures will always appear.

The capturing of the lattice structure’s surface is challenging because of its complex shape. There are three main approaches for it—image analyses, 3D optical measurement, and computed tomography. In the case of image analysis, an optical microscope is usually used to detect visible geometrical defects [2] and measure surface roughness [2,7] or dimensions in the strut’s cross-section [8,9,10]. The results are usually average values from several manual measurements, without complex information about the lattice structure morphology. Using 3D optical scanning allows for measuring dimensions with high accuracy (10^−3^ mm) [11,12] and, along with proper software tools, it also enables the automation of the data evaluation. However, this approach also has a limitation, in the case of complex geometries. The measurement can be performed only at the peripheral parts of the lattice structures; usually, they are not captured fully [11,13]. Computed tomography (CT) is generally used for internal porosity analysis [9,10,14,15]; however, this technology is applicable for strut geometry analysis, as well, as presented in several previous studies [11,16]. The main advantage of the CT approach is the ability to provide dimensional measurements over the whole volume of the complex structure [10].

Several authors have already performed dimensional analysis on the SLM lattice structures, which was most often used as an input for finite element analysis (FEA) geometry. The main disadvantage of these studies is a small number of measured samples, due to the complexity of the lattice structure and their common small dimensions. Suard et al. [17] used CT analysis to find the cylindrical equivalent for strut representation in FEA. His conclusion was confirmed by Vrana et al. [18], who achieved a good agreement between FEA and the experiment using Gauss cylinder. However, he also found that the actual cross-section of the manufactured lattice structures can be even more closely approximated by an ellipse, rather than the circle, and better compliance with mechanical behavior can be achieved using elliptical geometry. The authors also examined how to implement the actual shape of lattice structures into the FEA models and thereby improve the prediction of mechanical properties. Luxner et al. [19,20] used a model of geometry based on Timoshenko beam elements with quadratic interpolation functions, which made it possible to change the dimensions and shape of every single strut parametrically. However, a correction of the stiffness, in the vicinity of the vertices, was required. This was confirmed by Belardi et al. [21], who needed to use Young’s modulus increment for the ligament adjacent to the connections of open-cell foam nodes. The other advanced approach was presented by Ravari et al. [22], who used a script to create the combined lattice structure model using beam (B32) and solid elements (C3D10M). The script divided the lattice struts into equally spaced intervals, in order to enable a complex strut diameter change. Geng et al. [23] replaced the beam elements in a single unit cell of lattice structure with solid elements to balance computational efficiency and detailed results. The solid elements were used by several other authors, as well [17,18,24], who confirmed the suitability for the initial prediction of lattice structure behavior. Other authors used a combination of FEA and analytical models, which proved good compliance of results for linear elastic deformations, until the collapse of the structure [25].

The presented studies showed promising conclusions, resulting from complex geometry analysis, especially using CT technology. However, the conclusions are usually based on a small number of samples; typically, the dimensions of a few struts represent the whole structure. This study aims to achieve a deep investigation of the SLM manufactured lattice structures geometry using a micro CT measurement to capture and evaluate the whole geometry of the lattice structure in each strut (avg. *n* > 1000). As shown in the study [26], the dimensional deviations depend on the size of the struts; therefore, the lattice structures in the range of the strut diameter of 0.6–3.0 mm are evaluated. The results of the dimensional analysis are implemented into a new parametric FEA material model, driven by only one parameter, and compared with the experiment. Based on this study, the influence of the lattice structure dimensional deviations on its mechanical behavior is described and quantified via FEA—prediction using nominal vs. actual geometry.

## 2. Materials and Methods

The workflow of this study can be divided into three sections, i.e., SLM manufacturing of the lattice structures, µCT measurement and dimensional analysis, and FEA analysis and comparative quasi-static mechanical testing.

All lattice structure samples were produced in one SLM build job, in order to ensure the same manufacturing conditions. They were further digitized using µCT, and the dimensional analysis was performed in GOM Inspect software. Based on these results, the FEAs were prepared in Ansys Workbench 20.2. (Ansys, Inc., Canonsburg, PA, USA) software using CAD-designed and actual geometries of the lattice structures.

### 2.1. Lattice Structures Manufacturing by SLM

All the lattice structure specimens were manufactured from AlSi10Mg metal powder using the SLM machine (SLM 280 HL, SLM Solutions GmbH, Lübeck, Germany). The machine was equipped with a 400 W Ytterbium fiber laser (YLR) with Gaussian distribution, and the laser spot was focused on a diameter of 82 µm. During the SLM process, N_2_ atmosphere was used in the chamber, the oxygen level was kept under 0.2%, and the platform was preheated to 150 °C. The standard SLM process parameters, without a down-skin setup (SLM Solutions GmbH, Lübeck, Germany), were used for sample fabrication—laser speed (LS), laser power (LP), and laser focus (LF).

- Border laser paths: LS = 600 mm/s; LP = 300 W; LF= 0;
- Fill contour laser paths: LS = 555 mm/s; LP = 250 W; LF= −4;
- Hatch laser paths: LS = 1150 mm/s; LP = 350 W; LF = 0.

### 2.2. Lattice Structures Geometry

**Figure 1:**
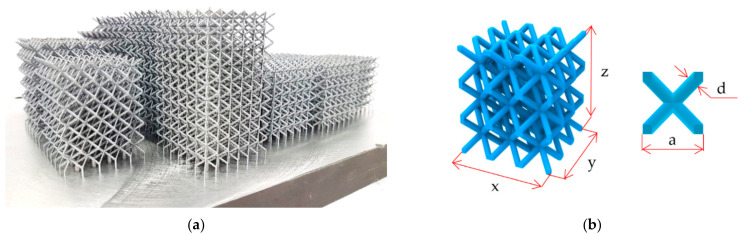
(**a**) The batch of SLM produced lattice structures cubes from AlSi10Mg. (**b**) Geometrical parameters of the lattice structures and a unit cell of BCC structure.

Lattice structure samples with body centered cubic (BCC) configuration were designed for evaluation of the shape and dimensional deviations and their influence on the mechanical properties. The main parameters defining the size of the lattice cubes (see Figure 1b) were the diameter (*d*) of the strut, size of the unit cell (UC) length (*a*), and relative density ($\rho_{r}$). Four of the specimens had a significantly larger height than the others to describe the changes of the deviations in the *z*-axis (build direction). Along with the samples for dimensional analysis, the samples for mechanical testing were also manufactured. Their mechanical properties were used to calibrate the AlSi10Mg material model in FEA. The parameters of all samples are shown in Table 1 and the batch of the samples after SLM manufacturing is shown in Figure 1a.

### 2.3. Lattice Structure Digitization

**Figure 2:**
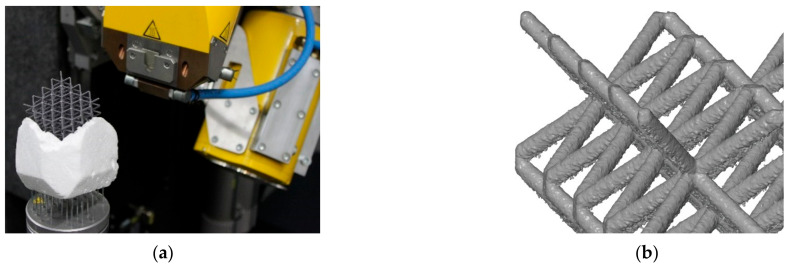
(**a**) µCT digitization process of one lattice structure specimen. (**b**) Output of the µCT digitization in the form of the polygonal mesh.

The lattice structures were digitized using µCT (GE phoenix v|tome|x L240, GE, Wunstorf, Germany), as shown in Figure 2a. The main used parameters of the X-ray tube were the voltage of 150 kV, current of 120 µA, and filter of 1.0 mm Sn plate. Each sample was digitized independently, in order to obtain results with the highest resolution. Therefore, the voxel size resolution was not uniform for all samples and, globally, was in the range of 40–52 µm, depending on the specimen’s maximum size. After the measurement, the data were reconstructed in the Datos reconstruction software, using the back-projection algorithm. All subsequent post-processing was performed in the software VGStudio MAX 3.1. The threshold for the evaluation of the surface was automatically set as a middle value between background and material peaks (ISO-50%) [27]. The base surface is further automatically improved, in such a way that the spots with the largest gradient changes are searched perpendicularly to the base surface contour (local thresholding). The output for the following analysis was a polygonal mesh, in the form of a STL file (see Figure 2b).

### 2.4. Compression Mechanical Testing

**Figure 3:**
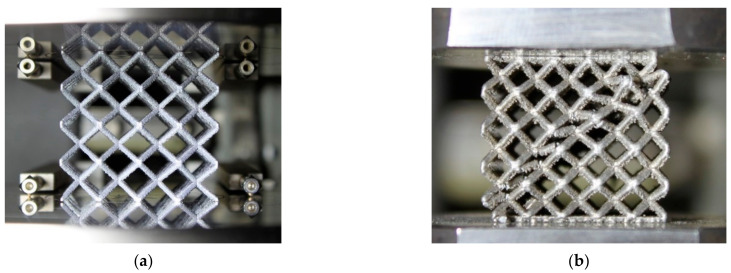
(**a**) The principle of the displacement measurement during the compression test. (**b**) Collapse phase of the BCC lattice structure during the compression test.

The universal machine for mechanical testing (Zwick Z250, ZwickRoell GmbH & Co. KG, Ulm, Germany) was used for the compression test of lattice structure cubes (Figure 3a). All samples, structures with strut diameters of 0.6 mm, 0.8 mm, 1.0 mm, and 1.5 mm, were pre-loaded with the force of 20 N and then loaded with a loading velocity of 2 mm/min (a standard value for tensile testing, which keeps inertia forces at a negligible level), until the progressive collapse of the lattice structure occurred (Figure 3b). Deformation fo the samples was measured using an extensometer to obtain accurate results, as is shown in Figure 3a. The outputs of the experiment, in the form of force reaction-displacement dependence, were also used for calibration of the FEA and evaluation of the other mechanical parameters.

### 2.5. Shape and Dimensional Analysis

The goal of the shape and dimensional analysis was to automatically obtain a full description of the manufactured lattice structure geometry, qualify it, and evaluate how this geometry differs from the original CAD model. The digitized surfaces were imported, together with the corresponding lattice structure’s CAD data, to the GOM Inspect Professional 2018 software (GOM GmbH, Braunschweig, Germany) for semi-automated analysis. This software offers a variety of tools for polygonal mesh processing and inspection. Inspection commands are accessible through the Python programming language module (gom) in the GOM Inspect software, and the commands can be used for the creation of automation scripts for evaluation.

**Figure 4:**
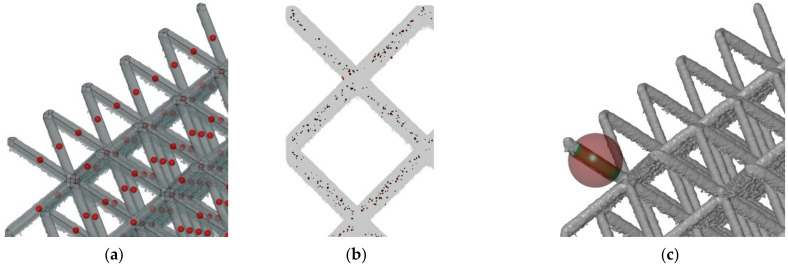

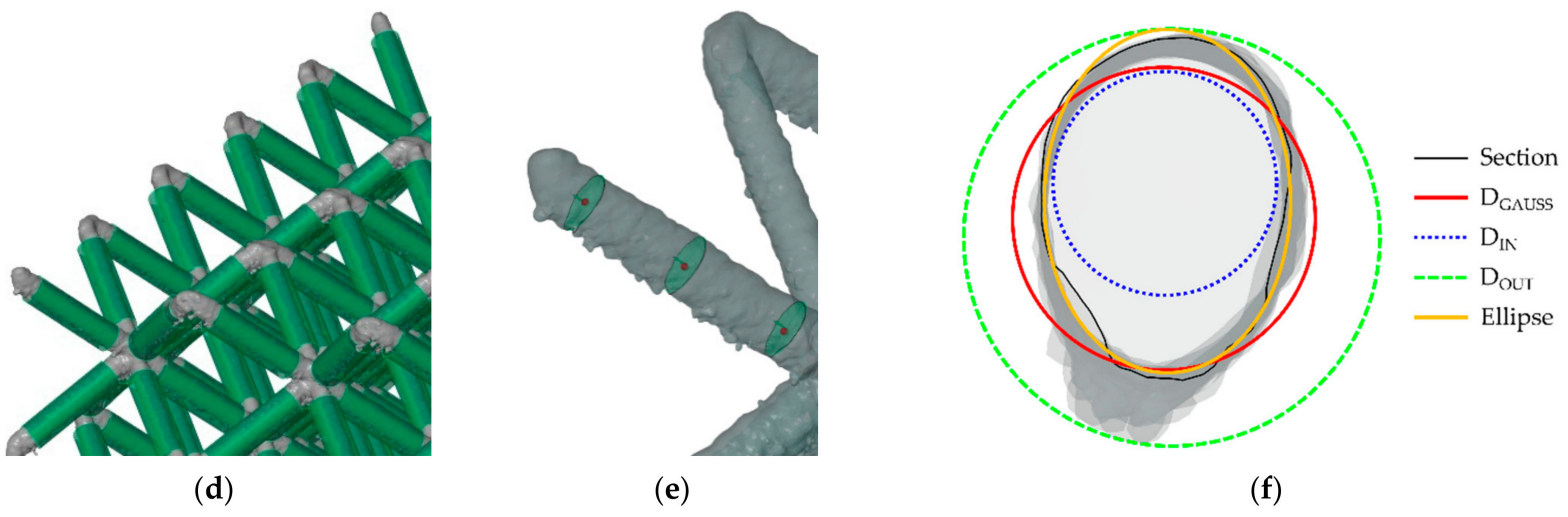
The procedure of lattice structure descriptive elements creation for the (**a**) polygonal mesh and CAD import; (**b**) deletion of internal voids; (**c**) strut’s data selection by sphere; (**d**) cylinders’ fitting; (**e**) points, planes, and sections creation and ellipses fitting; (**f**) dimensions evaluation.

The following steps describe, in detail, the process that leads to finding the shape and dimensional parameters of the manufactured lattice structures. The first four steps were performed manually, and the rest of them were performed automatically:

- Import—STL polygonal data of the digitized lattice structure and corresponding nominal CAD data (with strut’s midpoints; Figure 4a).
- Mesh processing—inner closed voids that remained in the structure after μCT analysis were deleted (Figure 4b). It allows for fitting the maximum inscribed cylinders into the struts.
- Alignment—the polygonal data were aligned to the CAD model by best-fit alignment.
- Lattice structure definition—the user was prompted to set the parameters defining the lattice structure (diameter of the strut, size of the unit cell, and number of cells in X, Y, and Z).
- Strut data selection—automatically for each strut. The CAD midpoints were used as a center of the selection sphere with a radius *r_s_* (1). The polygonal data inside this sphere were used for the further creation of the ideal cylinders. This procedure ensured comparatively the same area of selection, which is as large as possible for all lattice structure samples (Figure 4c). The different size of the lattice structure samples is driven by two main parameters, i.e., the size of the unit cell (*a*) and diameter of the strut (*d*). The sphere radius is then defined by the following equation:

(1) $$r_{s}=\frac{\frac{\sqrt{3}a}{2}-\left(2d+\frac{1}{d}\right)}{2},$$

- Cylinders’ fitting—previously selected data were used to fit three cylinders in each strut. The Gauss cylinder (D_GAUSS_)was created first, in order to find the axis of the strut. Then, the maximum inscribed (D_IN_) and minimum circumscribed cylinders (D_OUT_) were created on the same data selection, using the axis of the Gauss cylinder to ensure the same direction (Figure 4d).
- Points, planes, and sections—points were created at the ends of each Gauss cylinder axis, as well as in the middle of it. In each of these three points, a plane was created perpendicular to the cylinder axis. Subsequently, the planes were used to create the cross-sections and, thereby, obtain the 2D geometry of the strut (Figure 4e).
- Ellipses’ fitting—ellipses were fitted by Gaussian best-fit into each cross-section (Figure 4e).
- Dimensions evaluation—for each created element, dimensions were evaluated, i.e., the diameters of the cylinders and lengths of major/minor (referred to as E_MAJ_/E_MIN_) axes for the ellipses (Figure 4f).

After the automatic script computation, the data were exported to Microsoft Excel, in the form of CSV file, for further evaluation. Due to the numbering of the elements according to X, Y, and Z coordinates, their position can be determined exactly, and the results can be related to the position in the lattice structure.

### 2.6. Finite Element Analysis

The main goal of the FEA was to quantify the influence of the shape deviations of manufactured lattice structures on their mechanical properties. For this purpose, the numerical analysis was created in ANSYS Workbench R1, performing a quasi-static compression test of the BCC lattice structure. The three different types of geometry were compared—the lattice structures with a nominal circular cross-section and lattice structures with geometry based on the CT analysis, a Gauss cylinder (D_GAUSS_), and an elliptical (Gaussian best-fit) cross-section.

All types of geometries were created using tetrahedron elements with quadratic interpolation function (SOLID 187). The structures were further placed between two plates (SHELL 181) with linear elastic behavior of structural steel and artificially increased stiffness (almost rigid manners). This step was performed to reduce the simulation scene and computational effort. Mesh density was controlled by an automatic meshing algorithm embedded in ANSYS.

**Figure 5:**
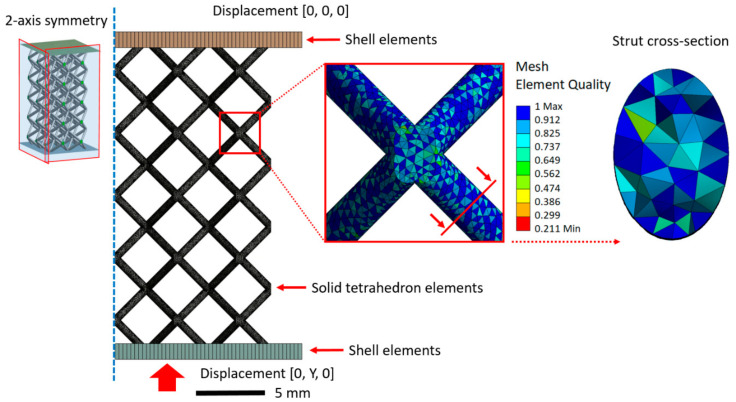
The meshed geometry of the lattice structure with boundary conditions.

Boundary conditions of the upper plate were set as fully constrained. The loading of the lattice geometry was applied through the bottom plate movement in the vertical direction (up to 2 mm in the *y*-axis direction). Between the lattice geometry and plates, frictional contact, with the coefficient of 0.15, was applied to prevent large translation. To reduce the computational time and hardware demands, a quarter symmetry model was used (see Figure 5).

**Figure 6:**
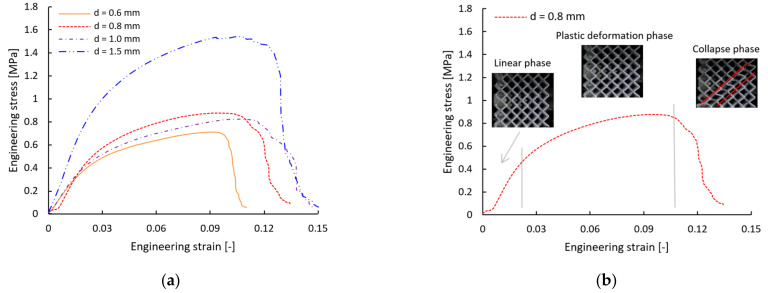
(**a**) Comparison of average stress–strain response of structures with different strut diameters. (**b**) Phases of structure deformation.

The bilinear elastic–plastic model of the AlSi10Mg material for lattice structures was chosen, based on the previous study [18]. The specific material parameters for various lattice structures’ strut diameters (Young’s modulus, tangent modulus, and yield strength) were defined according to the comparison of the FEA and four series of quasi-static compression tests (with nominal diameters 0.6, 0.8, 1.0, and 1.5 mm; Figure 6a).

## 3. Results and Discussion

### 3.1. Quasi-Static Compression Testing

The compression test was performed to determine the mechanical properties of the BCC lattice structures manufactured with different relative densities. Obtained data, given as force-displacement curves, were further evaluated to achieve an engineering stress–strain response (see Figure 6a). The engineering stress *P* was calculated as the acting force *F* divided by the cross-section area of the whole sample *S* (see Table 2).

In line with the expectations, the highest engineering stress was reached for the structure with the largest nominal strut diameter of 1.5 mm (almost 1.6 MPa), and the lowest values achieved structure with the smallest nominal strut diameter of 0.6 mm (approx. 0.7 MPa).

The mechanism of deformation was similar within all tested configurations of the BCC lattice structure (see Figure 6b). In the first phase, the structures were deformed without any visible fractures, and the engineering stress–strain curve was nearly linear (described by the compressive modulus of structure). The next phase was the non-linear region of stress–strain dependency (described by the tangent modulus of structure). The boundary between the regions was determined as 0.2% deformation, beyond the linear regime (yield stress of the structure). In the non-linear stage, visible plastic hinges in the near vicinity of diagonal nodes occurred. The stress and strain rose continuously, until the failure of these hinges became dominant, which caused the fracture of the struts and collapse of the structure.

### 3.2. Shape and Dimensional Analysis

#### 3.2.1. Influence of the Selection Sphere Diameter

As described in Section 2.5, a semi-automatic procedure was developed to analyze the dimensions of the lattice structure in each strut. As for the first parameter, the selection sphere size and its influence on the parameters of the created elements was tested to eliminate the possibility of results skewing. It was proven that, if this radius is not changed to extreme values (the sphere does not touch the node polygons), the effect of the radius is negligible. For example, for lattice structure #6, with a strut length of 9.5 mm, the radius of the selection sphere *r_s_* was altered between 1.9 and 3.1 mm, where the latter is the value calculated by Equation (1). The effect on the diameter of the Gauss cylinder and major ellipse axis was in the order of thousandths of mm (see Table 3). Therefore, the resulting values, presented further, are meaningful and free from systematic error, and they are calculated according to Equation (1).

#### 3.2.2. Full Sample Analysis on One Lattice Structure

**Figure 7:**
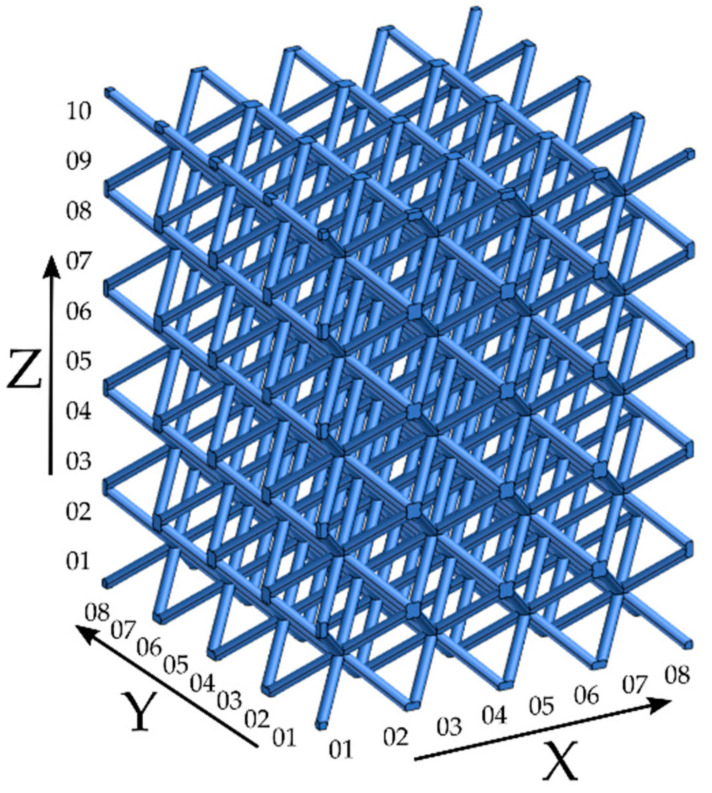
Numbering convention used in all structures.

For each specimen, the following summarization of the obtained results from the primitive elements fitting has been done. One specimen, #6–d1.25 mm strut diameter, was selected to present the common characteristics. Figure 7 represents the numbering convention used for all specimens. Z01 then stands for the first layer of struts, and Z10-Y08-X08 stands for the strut in the upper bottom right corner of the #6 structure.

**Figure 8:**
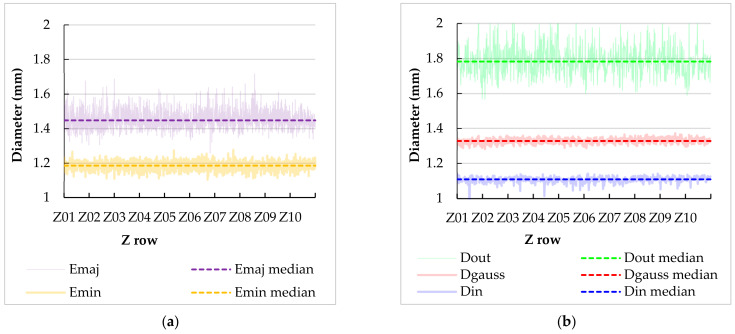
Values of strut characteristics of structure #6. (**a**) Major and minor axes of ellipses. (**b**) Minimum circumscribed, maximum inscribed, and Gauss cylinder’s diameters.

For each strut, five characteristics were evaluated—Gauss cylinder (D_GAUSS_), minimum-circumscribed cylinder (D_OUT_), maximum-inscribed cylinder (D_IN_), and the major (E_MAJ_) and minor (E_MIN_) ellipse axes. These characteristics were selected and used because they describe the real geometry of each strut. The graphs in Figure 8 represent the values for all struts in #6 specimen. The E_MAJ_ and E_MIN_ axes of ellipses were obtained as the average value of the three ellipses along each strut (Figure 8a). Same *y*-axes ranges were used to visualize the differences between these fitting elements. *x*-axes description denotes z rows, which are multiples of UC/2. In the particular case of #6, Z01 stands for z = 0 mm, Z02 for z = 5.5 mm, etc. 

The results in Figure 8 show that there are significant differences between the fitting elements, both in terms of median values and the spread of the results. The values in Table 4 summarize the results from the graphs into a simple numerical representation. Based on that, several general observations on individual types of elements can be made:

*Ellipse minor axis* represents the direction parallel to the build platform. It shows that the real struts are smaller than the nominal value in this direction. The explanation could be in the short circular laser paths during lattice structure production, as was described in the paper [28]. When the laser moves on the circular path, the outer area of the melt pool moves on a larger diameter than in the middle of the strut, the actual laser speed is higher, and the SLM input energy lower. It can result in a thinner laser track production and, finally, thinner diameter of the lattice structure strut. Moreover, there is also some visible spread of the values (displayed in standard deviation), showing flat sides of the struts that are not perfect.

**Table 4:** Median values and standard deviation for strut characteristics of structure #6.

Strut Characteristics	Median (mm)	Standard Dev. (mm)
E_MAJ_	1.446	0.052
E_MIN_	1.181	0.022
D_OUT_	1.724	0.087
D_IN_	1.117	0.020
D_GAUSS_	1.326	0.018

*Ellipse major axis* represents the direction perpendicular to the minor axis of the ellipse and includes the section through the up- and down-skin surfaces. It shows that the real struts are larger than the nominal value in this direction, caused by the weld deposits from the bottom of the struts. Additionally, there is an even larger spread of the values, showing that the weld deposits from the bottom are not uniform.

*Minimum circumscribed cylinder* represents an envelope around all artifacts on the strut. Its values are the largest of all of the elements. The large spread of values shows a high variability of diameters. This type of element has a low descriptive value, for the purpose of numerical or geometrical representation. However, the median value of the cylinders’ diameters can give an idea about the number of powder particles welded on the bottom side of the struts, and the standard deviation gives the idea of the roughness of the strut in the vertical direction.

*Maximum inscribed cylinder* represents the full material of the strut, without any protrusions or cavities (not considering any inner material voids in this µCT analysis). Its values are the smallest of all of the elements. The standard deviation is quite low, meaning that the diameter of the full material of the strut is relatively stable.

*Gauss cylinder* best represents the actual shape and dimension of the strut because most of the data, in which the cylinder is fitted, lies on the imaginary cylinder, and the diameter is less affected by outlier points. This is also confirmed by the lowest standard deviation of all the elements.

#### 3.2.3. Global Results

All of the structures were processed in the same manner as structure #6, and the results were obtained. Median values of diameters or axes lengths for all the structures are presented in Table 5.

**Figure 9:**
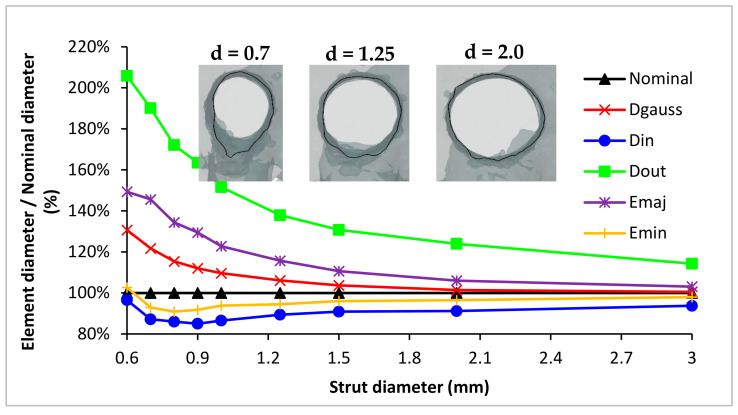
Graph of individual characteristic elements dimensions, relative to the nominal diameter, in relation to the nominal diameter of the strut.

For a better understanding of the results, a graph (shown in Figure 9) was created. It presents the percentage ratio of the size of characteristic elements, created on the actual geometry, to the nominal value of the lattice structure diameter and how this ratio changes with the increasing diameter of the strut. Examples of strut cross-sections for selected strut diameters are added to the graph for clarity.

**Figure 10:**
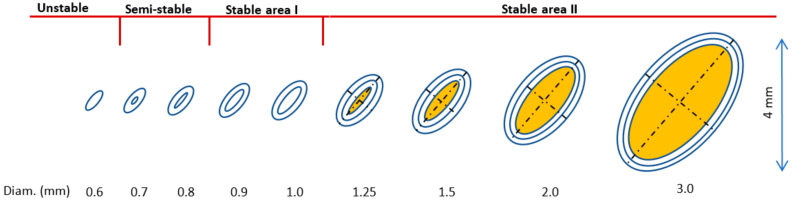
The schematic visualization of the used default SLM strategy.

From the presented results in Figure 9, it can be observed that:

- With the increasing strut diameter, the values of all characteristic elements approach the nominal value.
- With increasing strut diameter, the actual strut cross-section changes from an elliptical to circular.
- Based on the values, D_GAUSS_ and E_MIN_ are the closest representation of the real strut geometry to nominal CAD data. However, E_MAJ_ has a more similar course as D_GAUSS_.
- Percentual differences go up to 200% of the nominal size for the smallest strut diameter of the D_OUT_. On the other hand, D_IN_, for the same strut diameter, is almost the same as the nominal value.
- For the D_IN_ and E_MIN_, there is an interesting course. First, there is a decrease; then, from the diameter of 0.8 mm, there is an increase and approach to the nominal values. The explanation could be in the used laser strategy during the production of the thin lattice structure. Even if the default SLM strategy (SLM Solutions) was used, the thinnest struts were only produced by contour laser paths, due to the small cross-section. In addition, the hatch strategy was applied in the center of the strut for the struts with a larger diameter. In Figure 10, there is a schematic visualization of the used laser strategy. Up to a diameter of 0.6 mm, only one contour path was used. It caused an unstable SLM process, and remelted areas or gaps could occur in lattice structures, because the laser tracks overlap (OL) is changing with lattice diameter and not constant, as is necessary for stable SLM production. In a semi-stable area, the second path was added; however, the remelting occurred in the center of the lattice structure struts, due to the small diameter of the center contour. As the lattice structure dimensions were larger, the OL parameter and SLM process became stable (stable area I), until the hatch strategy was applied to the center of the strut (between diameter d = 1.0 and 1.25 mm). With the hatch strategy used, no more changes in the strategy occurred with increasing lattice structure diameter, and the process became completely stable (Stable area II). This transition area, with a strut diameters up to 1.0 mm and SLM laser strategy changes, could affect the lattice structure dimensional accuracy. The solution can be choosing only the contour laser strategy, as presented in the paper [28].

**Figure 11:**
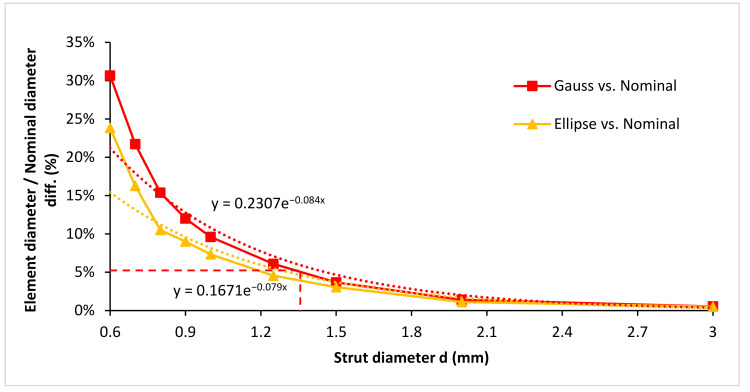
Graph of Gauss diameter and ellipse substitute diameter difference to the nominal diameter.

From the pictures and results in Figure 9, it is obvious that the strut’s cross-section area is most closely represented by the elliptic element. For better illustration, the elliptic shape, which is expressed by two parameters, E_MAJ_ and E_MIN_, was transformed to one circular parameter, *d_e_* in Figure 11. The chart presents the difference between the Gauss cylinder diameter D_GAUSS_ and ellipse substitute diameter *d_e_* to the nominal value, in relation to the strut diameter. Ellipse substitute diameter is expressed as:

(2) $$d_{e}=2\sqrt{\frac{E_{\mathrm{MAJ}}}{2}\cdot \frac{E_{\mathrm{MIN}}}{2}}$$

As it is evident from the chart in Figure 11, with increasing strut diameter, the deviation between both the Gauss cylinder D_GAUSS_ and substitute ellipse diameter *d_e_* from the nominal diameter is decreasing. From the strut diameter of 1.35 mm, the deviation of both of these diameters to the nominal diameter is less than 5% (indicated in the chart). Moreover, from the strut diameter of 0.8 mm, the deviation between D_GAUSS_ and *d_e_* is less than 5%. Based on these results, it can be concluded that, for strut diameters larger than 1.35 mm, the error is negligible with the use of nominal diameter for any further calculations. Additionally, for the strut diameters between 0.8 and 1.35 mm, the use of D_GAUSS_ is recommended, as it is much easier to obtain this value than *d_e_*, and the error is in the majority of cases negligible, as well. The trendlines of both graphs and their equations can be used to predict the diameter (Gauss and ellipse) for a specific nominal diameter and how this diameter would differ from the nominal one. The coefficient of determination for the trendlines is 0.96 for Gauss and 0.95 for ellipse. The trendlines do not well-represent the trend for the smallest diameters.

#### 3.2.4. Specific Results and Analysis of Phenomena

**Figure 12:**
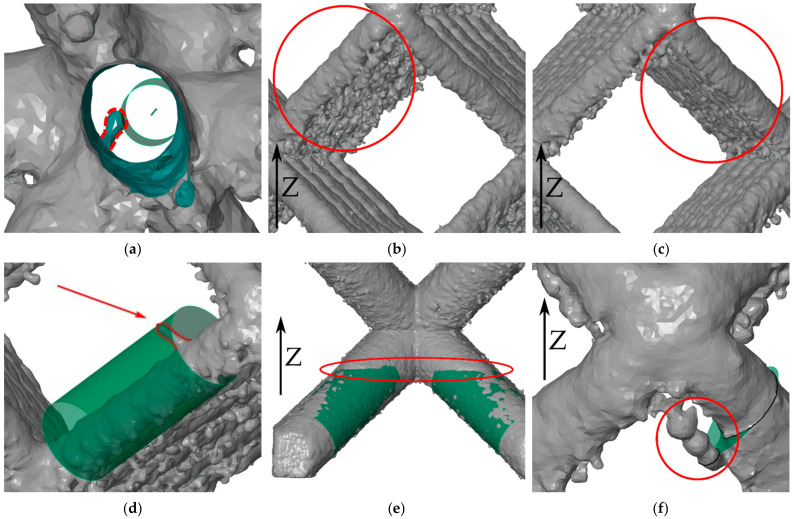
Illustration of lattice structures phenomena. (**a**) Protrusion pointing inside the strut (protrusion highlighted). (**b**,**c**) Different surface roughness on the down-skin surfaces. (**d**) Protrusion pointing outside the strut (protrusion highlighted). (**e**) Stair effect close to the unit cell node (a green Gauss cylinders are highlighting the stairs effect). (**f**) Weld deposits close to the unit cell node.

**Figure 13:**
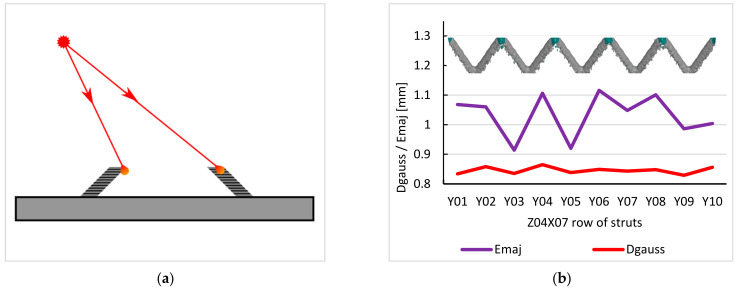
(**a**) Different direction of the laser source to each strut of the BCC unit cell and the resulting higher down-skin surface roughness on some of the struts. (**b**) Changes of the D_GAUSS_ and E_MAJ_ parameters over one row of struts in #2–d0.7 mm, showing a systematic variation of the surface roughness on the down-skin surfaces and proving the effect of the laser source to strut direction.

Besides the global results, which represent the overall characteristics of the lattice structures, several interesting phenomena have been observed in the results. Due to the numbering convention (described in Figure 7), the position of each element in a structure is known and could be retrieved. Following phenomena are presented on a particular structure (noted as e.g., #6–d1.25 mm), but some of them were also observed on other structures:

- Too small diameter of the maximum inscribed cylinder (#2–d0.7 mm) or too large diameter of the minimum circumscribed cylinder (#3–d0.8 mm)—significantly smaller or larger diameters than the median value were observed for D_IN_ and D_OUT_. It was found that this is caused by a protrusion pointing inside or outside the strut. Therefore, all results with significant deviations from the median value were manually checked, and it was confirmed that the values represent these protrusions (Figure 12a,d). However, because hundreds to thousands of struts were used for the calculation of the median value and only units of these protrusions were present in the structures, the resulting median value should only be influenced to a minimal extent, and these values were not filtered out. The situation shown in Figure 12d could occur due to splashing of the melt pool in the liquid phase or during powder recoating, as described in [29]. The situation shown in Figure 12a could also occur as a result of the melt pool splashing or as a large opened under-surface pores.
- Different roughness on the down-skin surface of the neighboring struts (#2–d0.7 mm)—this is caused by non-uniform weld deposits from the bottom surface of the struts. The non-uniformity is dependent on the BCC struts’ orientation to the laser source and distance between the laser and produced lattice structure. Therefore, sample #2–d0.7 mm, which was placed in the corner of the platform, was used to illustrate the effect. Within the BCC unit cell, the orientation of each strut, with respect to the laser direction, is different (Figure 13a). It influences the surface roughness on the bottom surface of the struts (Figure 12b,c), and the resulting values of the D_GAUSS_ diameter change over the structure (Figure 13b). This effect was also partially described on nine samples (hollow twelve-sided frustum of a pyramid) in the study [30].

- Stairs effect close to the unit cell node (#9–d3 mm)—this effect is most evident in the lattice structures with the largest diameters. From the dimensions of the ellipse lying on the upper end of the strut, as well as from Figure 12e, there is an apparent stair effect causing the increase of the E_MAJ_. The stair is approximately in the height of contact of the struts (Figure 12e). From this fact, it can be deduced that the stair effect is caused by the thermal stresses during the solidification of the first node layers, which bend the free ends of the struts into the node.
- Significant differences among the major ellipse axes for smaller diameters (#2–d0.7 mm)—in some cases (e.g., Z04-Y04), this characteristic changes in a regular pattern; in other cases, it is not so regular. This is due to the weld deposits close to the unit cell node, through which the section passes (Figure 12f), and it may also be a result of powder recoating, as described in [29].

These detailed results, which were obtained through the depth analysis of large data using µCT and semi-automated script, indicate significant local instabilities in the SLM production process. Therefore, it is always necessary to analyze a statistically significant number of values, in order to describe the dimensions of the whole lattice structure. Individual values may not represent it well.

### 3.3. Finite Element Analysis

Within the FEA, both previous analyses were directly used. The results of the shape analysis were used for the preparation of an actual geometry of the lattice structures using elliptical and circular strut cross-sections. The results of a quasi-static compression test were used for calibration of the AlSi10Mg elastic–plastic model of the material.

#### 3.3.1. Model of the AlSi10Mg Lattice Structures

**Figure 14:**
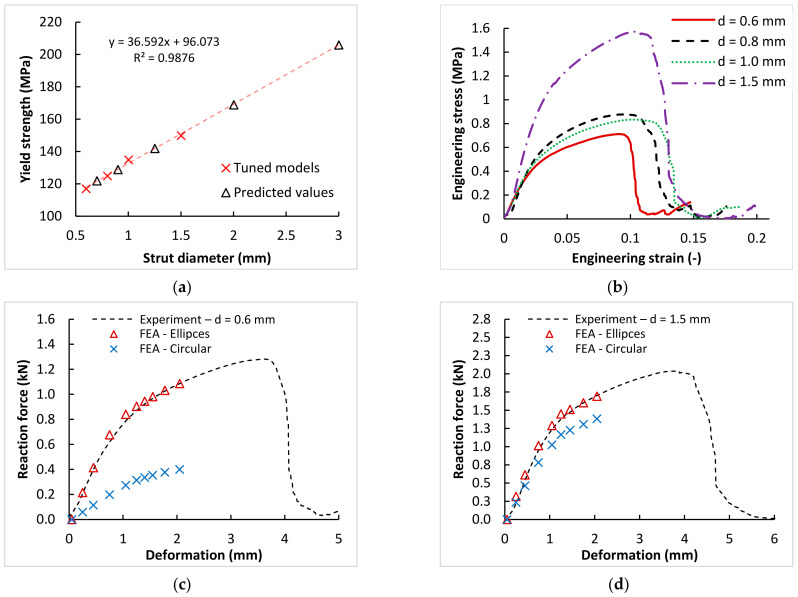
(**a**) Definition of the variable parameter YST, based on the nominal lattice structure diameter. (**b**) The results of the mechanical test used for calibration of the FEM models. (**c**) Comparison of the experimental and calibrated FEM results on thin lattice structure (*d* = 0.6 mm). (**d**) Comparison of the experimental and calibrated FEM results on thin lattice structure (*d* = 1.5 mm).

The default bilinear elastic material model of aluminium alloy, from the Ansys material library, was used for the initial calculations in this study. The reverse FEA was performed using this default material model and the actual geometry of mechanically tested samples to compare the experimental and FEA results. The reversed FEA showed different deviations, depending on the nominal dimensions of the lattice structures. Based on that, the parameters of the material model were tuned to achieve compliance with the testing force-deformation curves (triangular marks in Figure 14c,d). This calibration approach showed the following results: the parameters as Young’s modulus and tangent modulus are not dependent on the dimensions of the lattice structure (can remain the same for all geometries, i.e., E = 28 GPa, Et = 2 400 MPa), and the yield strength parameter (YST) increases linearly with the nominal diameter of the lattice structure (Figure 14a). The resulting YST values were interpolated with a linear function (for structures with nominal strut diameters of 0.6 mm, 0.8 mm, 1.0 mm, and 1.5 mm). Based on the interpolation function, the YST values for other strut diameters were suggested (Equation (3)). All parameters of the material model for BCC (BL-I) and plates (BL-II), as defined in Ansys, are in Table 6.

YST = 36.592 *d* + 96.073 (3)

#### 3.3.2. The Deviation in Mechanical Properties between Nominal and Actual Shape

The FEA using the Al-EP2 material model allowed us to quantify the deviations in actual (elliptical cross-section) and planned (nominal cross-section) lattice structure mechanical behavior. As the main parameters for comparison, the global mechanical parameters of the whole lattice structure Young’s (E_s_) and tangent (E_ts_) moduli were used. Lattice structures with strut diameters up to 3 mm were analyzed. The boundary conditions of the performed FEAs corresponded to the previous compression test.

**Figure 15:**
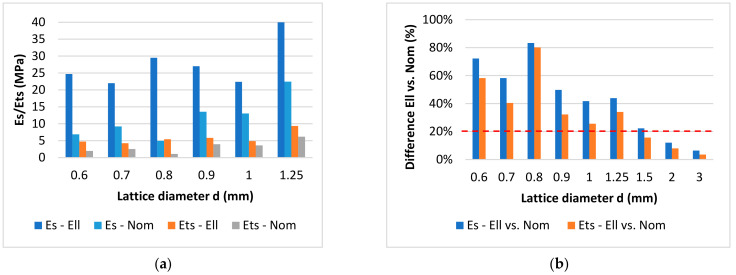
(**a**) Comparison of average stress–strain response of lattice structures with different strut diameters, up to the nominal diameter of 1.25 mm. (**b**) Comparison of the relative deviations of the nominal circular cross-section (Nom.) and actual elliptical cross-section (Ell.).

The complete results are shown in Table 7, as well as in Figure 15. They very well correspond with the results of the shape and dimensional analyses. As shown below, the mechanical deviations are large mainly on the lattice structures with small diameters. In the case of the diameter of 0.8 mm, the deviations of both moduli were around 80% and, in the case of 0.6 mm, around 70% of Young’s modulus. The deviations decreased as the diameter increased. Above the diameter of 1.0 mm, the deviations were around 40% and, above the diameter of 1.5 mm, around 20% or even less. In Figure 15a, the results are compared in absolute values only, up to 1.25 mm, for better chart readability. The relative comparison of all FEA results is shown in Figure 15b.

**Figure 16:**
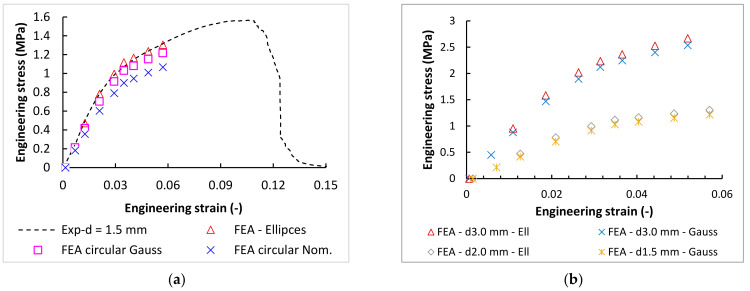
(**a**) The comparison of the experimental data and FEA with three types of cross-section (elliptical, circular Gauss, and circular nominal). (**b**) The comparison of the FEA results for the lattice structure dimensions of 2.0 and 3.0 mm.

From the results presented in Figure 15b, the imaginary boundary can be found around values for the nominal strut diameter of 1.5 mm. From the point of view of the dimensional and numerical simulation, this seems to be the limit from which the lattice structure is well-represented by a circular cross-section. The control calculations were performed for this limit diameter of 1.5 mm. As shown in Figure 16a, the Gauss cylinder is very close to the experimental and elliptical results. The result for the diameters of 2.0 and 3.0 mm are shown in Figure 16b. The deviation is below 10% in both cases (Figure 15b). This confirmed the joint conclusion of both analyses, and the dimensions of 1.5 mm could be the limit between elliptical and circular representation in FE analyses.

## 4. Conclusions

This study deals with geometrical and dimensional deviations of the lattice structures manufactured by SLM technology, resulting from the manufacturing process itself. Moreover, it addresses the influence of these deviations on the mechanical properties of such lattice structures, in terms of stress–strain response, and introduces a universal FEA material model that is applicable to a range of strut diameters.

In this study, a set of lattice structured samples, with nominal strut diameters ranging from 0.6 to 3.0 mm, was produced using SLM technology. These samples were digitized using computed tomography (µCT) and further analyzed using a semi-automatic procedure. Thanks to this approach, a large number of dimensional characteristics were obtained; moreover, anomalies associated with the SLM process were identified. The evaluation of individual primitives has been described step-by-step and can be replicated, either for the same structure (BCC), but different material, or with minor modifications, as well, for other types of structures. The proposed procedure is not significantly dependent on the size of the evaluated area, as has been experimentally verified.

The main conclusions of this study follow:

- Significant ellipticity was found on the lattice structure with the thinnest struts. The increased ellipticity is mainly caused by weld deposits on down-skin surfaces, resulting from the manufacturing process. The ellipticity decreased with increasing diameter (Figure 9).
- The obtained struts’ cross-section dimensions (Gauss, ellipse) were interpolated by exponential function, in the range of 0.8 to 3.0 mm (Figure 11). This function allows for predicting the expected cross-section dimensions for FEA simulation for any nominal diameter among this range.
- The unexpected deviations were observed on the thinnest lattice structures, with a diameter up to 0.8 mm. It is caused by the change of the laser strategy from contour to contour-hatch within the default SLM parameters. The solution may be to use the contour laser strategy, as presented in the paper [28].
- Using FEA reverse approach, Al-EP2 material model of the AlSi10Mg lattice structure was developed for the range of strut diameters from 0.6 to 1.5 mm. This material model includes the constant material parameters (especially Young’s and tangent moduli), as well as variable parameter yield strength (YST), related to the nominal diameter of the used lattice structure (Figure 14).
- The FEA confirmed the significant effect of the ellipticity of the thinnest struts on the mechanical properties of the lattice structures. It also made it possible to quantify the deviations between the actual and nominal lattice structure mechanical properties. (Figure 15).
- Both analyses, i.e., the shape and dimensional analysis (SDA) and FEA, identified the lattice structure diameter of around 1.5 mm as the limit value. Within SDA, the strut’s ellipticity is already not significant from this diameter, and the structure is well enough represented by the Gaussian cylinder (Figure 11). Within FEA, the difference in mechanical properties is below 20% (Figure 15).

## Figures and Tables

**Table 1:** The parameters of the BCC lattice structure batch.

#	Nominal Lattice Diameter *d* (mm)	Size of the UC *a* (mm)	CAD Relative Density $\rho_{r}\;(\%)$	X and Y Length (mm)	Z Height (mm)	X and Y Number of UC	Z Number of UC	Number of the Struts *n* (-)
1	0.6	7	3.69%	42	70	6	10	2880
2	0.7	8	3.84%	40	48	5	6	1200
3	0.8	8	4.95%	40	72	5	9	1800
4	0.9	9	4.95%	45	54	5	6	1200
5	1.0	10	4.95%	40	70	4	7	512
6	1.25	11	6.61%	44	55	4	5	512
7	1.5	12	7.55%	36	72	3	6	216
8	2.0	13	11.10%	39	52	3	4	288
9	3.0	15	17.85%	45	60	3	4	216

**Table 2:** The results of the experimental mechanical testing of BCC lattice structure cubes.

#	Nominal Lattice Diameter *d* (mm)	Max Standard Force *F_max_* (N)	Deformation in *F_max_* *x* (mm)	Max Engineering Stress *P_max_* (MPa)	Strain in *P_max_* (-)
1	0.6	1279	3.98	0.73	0.09
2	0.6	1256	3.82	0.71	0.09
3	0.6	1281	3.63	0.73	0.09
4	0.8	1407	3.66	0.88	0.09
5	0.8	1403	3.84	0.88	0.10
6	0.8	1398	3.65	0.87	0.09
7	1.0	1336	4.20	0.83	0.11
8	1.0	1205	6.30	0.75	0.16
9	1.0	1319	4.27	0.82	0.11
10	1.5	2039	3.80	1.57	0.11
11	1.5	1999	3.85	1.54	0.11
12	1.5	2029	3.81	1.57	0.11

**Table 3:** Influence of the selection sphere size (lattice structure #6).

Radius of Selection Sphere *r_s_* (mm)	3.1	1.9
Gauss cylinder D_GAUSS_ (median) (mm)	1.328	1.326
Ellipse major axis E_MAJ_ (median) (mm)	1.185	1.181

**Table 5:** Median values of diameters (axes lengths) for all the fitting elements of all structures.

#	Nominal Lattice Diameter *d* (mm)	Gauss Diameter D_GAUSS_ (mm)	Max. Inscribed D_IN_ (mm)	Min. Circumscribed D_OUT_ (mm)	Ellipse Maj. Axis E_MAJ_ (mm)	Ellipse Min. Axis E_MIN_ (mm)
1	0.6	0.784	0.579	1.235	0.896	0.616
2	0.7	0.852	0.610	1.331	1.019	0.650
3	0.8	0.923	0.688	1.377	1.075	0.727
4	0.9	1.008	0.765	1.471	1.165	0.826
5	1.0	1.096	0.865	1.516	1.228	0.938
6	1.25	1.326	1.117	1.724	1.446	1.181
7	1.5	1.555	1.363	1.961	1.659	1.440
8	2.0	2.028	1.823	2.479	2.120	1.929
9	3.0	3.016	2.812	3.428	3.093	2.939

**Table 6:** Material model parameters used for lattice structures’ FEA.

Parameters	BL-I (BCC)	BL-II (Plate)	Unit
Density	2680	7850	kg·m^−3^
Young’s modulus	28,000	2 × 10^14^	Pa
Poisson’s Ratio	0.33	0.3	-
Bulk modulus	2.745 × 10^10^	1.666 × 10^14^	Pa
Shear modulus	1.053 × 10^10^	7.692 × 10^13^	Pa
Yield strength	Equation (3)	-	MPa
Tangent modulus	2400	-	MPa

**Table 7:** The complete results of FEAs, using an elliptical and circular (nominal) cross-section of the lattice structure.

Nominal Strut Diameter *d*	Elliptical Cross-Section		Circular Cross-Section		Difference Ell vs. Nom.			
	E_s_—Ell	E_ts_—Ell	E_s_—Nom	E_ts_—Nom	E_s_		E_ts_	
(mm)	(MPa)	(MPa)	(MPa)	(MPa)	(MPa)	(%)	(MPa)	(%)
0.6	24.7	4.7	6.9	2.0	17.8	−72%	2.8	−58%
0.7	22.0	4.3	9.2	2.5	12.8	−58%	1.7	−40%
0.8	29.5	5.4	4.9	1.1	24.6	−83%	4.4	−80%
0.9	27.0	5.8	13.6	4.0	13.4	−50%	1.9	−32%
1	22.4	4.9	13.1	3.6	9.4	−42%	1.2	−26%
1.25	40.0	9.4	22.5	6.2	17.5	−44%	3.2	−34%
1.5	42.3	8.2	32.9	6.9	9.4	−22%	1.3	−16%
2	93.0	18.4	81.8	17.0	11.2	−12%	1.5	−8%
3	278.4	59.9	260.6	57.8	17.8	−6%	2.1	−4%

## Data Availability

Not applicable.
